# Selective Removal of Neutrophil Extracellular Traps (NETs) Combined with Ex Vivo Lung Perfusion (EVLP): Current Evidence and Future Perspectives

**DOI:** 10.3390/jcm14228136

**Published:** 2025-11-17

**Authors:** Anton Sabashnikov, Sanjay Agrawal, Bartlomiej Zych, Ihor Krasivskyi, Syed Hussain Abbas, Dengu Fungai, Thomas Williams, Louit Thakuria, Andrew Aswani, Mohamed Osman, Maria Monteagudo-Vela, Vasiliki Gerovasili, Anna Reed

**Affiliations:** 1 Guy’s & St Thomas’ NHS Foundation Trust Royal Brompton and Harefield Hospitals Harefield Hospital, Hill End Road, Harefield UB9 6JH, UKmariamonteagudo.ela@gmail.com (M.M.-V.);; 2 Department of Cardiac Surgery, University Hospital Bonn, 53127 Bonn, Germany; 3 Nuffield Department of Surgical Sciences, University of Oxford, Oxford OX1 4BH, UK

**Keywords:** neutrophil extracellular traps (NETs), ex vivo lung perfusion (EVLP), lung transplantation

## Abstract

Severe discrepancy between availability of donor organs suitable for clinical transplantation and the proportion of patients on the waiting list has resulted in several clinical problems. First, waiting times for a suitable organ match have become increasingly long, leading to higher mortality while awaiting transplantation. Second, to address this issue, more “marginal” donor lungs have been used in the last two decades, inevitably leading to higher risk of perioperative and long-term complications. The ex vivo lung perfusion (EVLP) technology has been used to recondition marginal donor organs for clinical transplantation. There remains a further untapped pool of donor organs that are currently deemed too injured even for reconditioning via currently available EVLP strategies and are therefore discarded without reconditioning attempts. As the clinical use of EVLP has reached its full potential, further adjunct technologies, such as selective NET removal, cytokine removal and cell therapy techniques, may improve reconditioning outcomes and lead to increased number of donor organs transplanted. Moreover, NET removal may significantly improve donor organ quality and, therefore, the outcomes of recipients after lung transplantation. Such adjunct technology may also provide short- and longer-term benefits in reduction in early graft failure (primary graft dysfunction, PGD) and longer-term chronic lung allograft dysfunction (CLAD, previously known as chronic rejection) via more favorable early immune priming of organs. In this article we present current evidence and future perspectives on this novel intervention strategy that can be used on human donor lungs with the view to increase the utilization rate in lung transplantation in the near future.

## 1. Introduction

Lung transplantation remains the gold standard therapy for end-stage lung disease refractory to medical treatment. There has been a severe discrepancy between the number of donor organs available and the number of patients requiring lung transplantation over the last two decades. Despite significant improvements in preservation techniques, donor organ quality has increasingly become more marginal, and the utilization rate remains very low. After careful evaluation following organ assessment and retrieval, a significant proportion of organs is considered not suitable for transplantation and/or reconditioning using normothermic ex vivo lung perfusion (EVLP) and must be discarded [1]. To address this problem, further technical advances are needed to improve donor lung quality by reconditioning approaches and to increase the number of donor lungs that may become transplantable. A focused table summarizing key experimental models, techniques, and outcome improvements, which have already been implemented in preclinical research, is summarized in Table 1. In brief, due to a large number of research studies on EVLP, they can be classified into (1) projects targeting extended preservation and functional assessment of donor lungs; (2) representing platforms for evaluation and rehabilitation of marginal donor lungs; and (3) models for lung-injury-specific treatments [2] Additionally, several specific trends have been observed in recent years, representing new sophisticated approaches to EVLP, such as implementation of dual EVLP for selective perfusion of both pulmonary and bronchial arteries [3,4], research on specific biomarkers to predict survival and to monitor therapeutics during EVLP [5], and delivery of specific therapies to reduce ischemia–reperfusion injury (IRI) and inflammation [6,7,8,9,10]. NET removal from donor lungs on EVLP represents a novel conceptual approach and has large potential both for an increased number of transplantations and improved outcomes in recipients after lung transplantation.

## 2. Scientific Background

EVLP has emerged as a clinical tool for the assessment and reconditioning of human lungs for potential clinical transplantation. A prospective multicenter study comparing transplant outcomes between EVLP and standard donor lungs involving all five UK officially designated NHS adult lung transplant centers (DEVELOP-UK), including our institution, showed that only one-third of donor lungs subjected to EVLP were successfully resuscitated and transplanted [2]. Moreover, the EVLP group was associated with an increased rate of primary graft dysfunction (PGD), higher requirement of extracorporeal membrane oxygenation (ECMO) and lower survival over 12 months; however, the data were consistent with no difference in survival between groups. EVLP limitations in terms of successful resuscitation potential of marginal donor lungs were particularly evident for donation after circulatory death (DCD) organs revealed in a recent large United Network for Organ Sharing database analysis [3]. However, high-volume experienced EVLP centers, such as the Toronto Lung Transplant Program, were able to achieve greater results both in terms of utilization rate of organs resuscitated on EVLP and also outcomes after transplantation after EVLP assessment [4]. Further adjunct organ management approaches on EVLP, such as advanced diagnostic assessment of allografts and the administration of advanced therapy to attenuate organ injury and to immunomodulate allografts, have been recently proposed, however not broadly implemented into clinical practice [5]. Over the last two decades, several technological advancements have been implemented to improve the functional assessment of donor lungs, including development of superior perfusion solutions, use of artificial intelligence (AI), and integration of gene therapy [6]. Nevertheless, there is still a large potential for improvement in the basic EVLP approach, i.e., by using sophisticated and adjunct technology that could significantly contribute to improvement in resuscitation outcome and subsequent outcome in recipients receiving such donor lungs.

NET are well studied web-like chromatin and mitochondrial DNA structures, including histones and granule-derived antimicrobial proteins, such as MPO (myeloperoxidase), and NE (neutrophil elastase). They are released extracellularly by neutrophils as part of the antimicrobial defense reaction [7]. However, some clinical scenarios may facilitate overproduction of NET leading to their negative impact, including severe inflammation, oxidative, cardiovascular and neurodegenerative stress, potentially causing tissue and organ damage [7,8]. Pathologic NET overproduction has been studied in several clinical conditions, such as diabetes, atherosclerosis, occlusive diseases, autoimmune disorders, cancer, and severe viral infections [9,10,11]. Moreover, overexpression of NET was shown to be associated with worse postoperative outcomes following human lung transplantation [12]. To address this issue, several advantages of NET removal have already been shown in animal experimental research on lungs with initiated acute lung injury (ALI), which is clinically similar to findings in lung allografts following donation [13]. However, there have been no experimental studies examining NET removal from human allografts. Such research is vital to provide evidence on effectiveness of NET clearance and its positive effect on allograft function, which can be evaluated using the EVLP platform. The ultimate goal of NET removal methodology is to increase the number of potentially transplantable lungs and to improve donor organ quality for better outcomes in recipients after lung transplantation.

One of the recent focuses in research on lung transplantation with and without EVLP use has been the role of NET on outcomes after lung transplantation. It was particularly shown that elevated levels of NET both in EVLP perfusate and in recipients after lung transplantation were closely associated with severe PGD [12,13,14]. Recent animal experimental research showed that removing NETs during EVLP effectively improved pulmonary function and morphology in aspiration-damaged donor lungs [13]. While there are a few animal experimental studies published on selective NET removal in conjunction with EVLP, translation of this research on humans remains the merit of further investigations. Also, previous animal studies were performed on lungs that were subjected to induced ALI. While ALI and acute respiratory distress syndrome (ARDS) are clinically similar to findings in a significant proportion of marginal donor lungs, injured donor lungs may be associated with a broader spectrum of pathophysiological, biochemical and immunological changes beyond “typical” ALI. Therefore, the ability to remove NETs during EVLP could represent a new therapeutic approach for lung transplantation expanding the donor pool, decreasing mortality on the waiting list and improving clinical outcomes of recipients after lung transplantation.

## 3. Current State of Research and Future Perspectives

The increasing number of explanted organs that were sustained on EVLP, both for clinical and research purposes, allowed for an increased utilization rate as well as for establishing a platform for ongoing research using whole human lungs that were sustained outside of the body [15,16,17]. As the clinical use of EVLP has reached its full potential, further adjunct technologies, such as selective NET removal, may significantly improve reconditioning outcomes and lead to an increased number of donor organs transplanted and improve recipients’ outcomes [16,18]. Furthermore, this technology may allow for potential use of injured organs currently deemed unsuitable for transplant, even using currently available clinical reconditioning approaches on EVLP [18].

It is well known that circulating NETs are established within human ALI and ARDS within critically unwell patients with sepsis, and their selective removal in this context shows promise for therapy [19]. Within human lung transplant, there is a lack of literature regarding the role of NETs in graft injury; however, one group demonstrated that higher levels of NETs within the perfusate of lungs supported on EVLP were associated with worse outcomes after transplant, and another showed that NETs are pathogenic in both clinical and experimental models of primary graft dysfunction (PGD) after lung transplant [20,21]. One of the most promising animal studies on NETs in experimental lung transplantation recently published by Mittendorfer et al. showed that removing NETs may represent a solid therapeutic strategy to improve donor organ function during EVLP reconditioning. Using this adjunct technology, this group was not only able to significantly improve PaO_2_/FiO_2_ ratios in lung allografts, decrease levels of cell-free DNA, extracellular histones and proinflammatory cytokines in EVLP perfusate but also improve histopathological characteristics, such as less immune cell infiltration and edema.

Despite promising evidence shown in animal settings, translation of this research to humans merits further investigation. There is further need to prove the efficiency of selective NET removal in real-world scenarios, where a wide spectrum of pathophysiological, biochemical, and immunological changes in marginal donor organs can be targeted by this novel approach [17]. However, to proceed with consideration of intervention, such as removal of NETs and their components in human donor lungs, the first step of developing this strategy is to establish laboratory-based techniques and NET quantification to outline organ injury and recovery profiles in human lungs sustained on EVLP. This will allow for baseline assessment of clinical physiological parameters, injury biomarkers and NET burden during therapeutic intervention [20].

## 4. Marginal Donor Lungs and Potential to “Safely” Push the Limits

The baseline assessment of the donor lung usually starts during organ retrieval and includes chest X-ray, bronchoscopy, PaO_2_/FiO_2_ ratio measured at FiO_2_ 100% and PEEP of 5 mm H_2_O, other ABG parameters, standard laboratory parameters including inflammatory markers, liver function tests (LFTs), renal function tests (RFTs), hematology and coagulation profiles. Respiratory physiotherapy using a ventilator-based maneuver to remove intrabronchial secretions is applied, if clinically indicated, during the donor management process. Also, a manual clinical assessment of the lung tissue quality (presence of palpable masses, blebs or bullae, signs of volume overload or consolidation), manual removal of atelectasis and measurements of selective ABGs from each pulmonary vein are performed. In a substantial proportion of cases, several assessment parameters appear abnormal, meeting criteria for marginal allografts that were shown to be associated with poorer outcomes, if transplanted directly [22]. While some of the marginal donor organs may be subjected to a conventional EVLP assessment and potentially transplanted, a substantial number of allografts are still discarded at the time of retrieval or later after unsuccessful EVLP resuscitation attempts [23]. Therefore, selective NET removal may not only improve resuscitation potential of marginal allografts but also potentially make currently completely non-transplantable organs transplantable. Importantly, pushing the limits in accepting allografts with very poor lung function for EVLP and selective NET removal will still be a “safe” approach as no organ will be transplanted into the recipient until a thorough evaluation of organ function post-intervention shows acceptable results. Also, removal of NET from donor lungs that are currently “only” subjected to conventional EVLP treatment may inevitably improve donor lung function, resulting in better outcomes after lung transplantation.

## 5. Specifics in Allograft Assessment During and After Selective NET Removal

In addition to conventional physiological and hemodynamic evaluation of allograft function during the standard ELVP resuscitation process, further emerging molecular and immunological assessment strategies can be used in conjunction with selective NET removal.

### 5.1. Conventional Physiological Evaluation

Over the last two decades, standard acceptance criteria during EVLP runs have been limited to delta pO_2_ ≥ 350 mmHg, stable or decreasing pulmonary and airway pressures and pulmonary compliance, as well as acceptable perfusate loss. While it is common practice and, in most cases, conventional physiological assessment may sufficiently predict organ transplantability, a deeper and more detailed evaluation in terms of molecular and immunological changes, particularly in conjunction with selective NET removal, might be of high importance to foresee potential risks for PGD or even long-term complications, such as chronic lung allograft dysfunction CLAD targeting improved overall outcomes in recipients [15,16,24].

### 5.2. Histopathological and Molecular Analysis

Assessment can include exhaled breath gas sampling for the measurement of ethylene, nitric oxide, carbon monoxide, acetaldehyde, amongst others. Exhaled breath condensate (EBC), bronchoalveolar lavage fluid (BALF), perfusate and tissue biopsy samples can also be collected for analysis.

As previously shown in animal models, to assess efficiency of selective NET removal technology, molecular analysis can evaluate levels of citrullinated H3 in tissue, and extracellular H3 nucleosomes, cell-free DNA, total extracellular histone and extracellular H3 nucleosome in plasma. Also, citrullinated (Cit)-H3 in tissue can be additionally assessed and quantified on imaging. Fibrin depositions can be quantified by immunohistochemistry, and the extent of fibrin depositions in lung tissue can be presented on imaging and quantified using a fibrin score. Immune cell marker allograft inflammatory marker (AIF)-1 and proinflammatory cytokines can also be measured during the EVLP run. Moreover, multiple omics analyses such as genomics, transcriptomics, immunomics and proteomics may provide further benefits. Samples should be interrogated for the impact of NET formation within both the systemic (perfusate) and tissue (bronchoalveolar lavage and lung biopsy) compartments to provide a full spectrum of assessment. Histological examination may also include electron microscopy and novel imaging methods. Isolated cell preparations may also be studied for their in vitro responses to injury by establishing a primary cell culture line.

### 5.3. Digital Imaging

The lungs can be assessed radiologically, whilst the organs are sustained on ex vivo perfusion using X-ray, CT scan, or other forms of nuclear imaging. Also, digital photographs and videos can be recorded, whilst the organs are sustained ex vivo.

This profound combined analysis will help understand the differing phenotypes of lung injury and health when analyzed in conjunction with clinical physiological parameters such as gas exchange, compliance, and pulmonary vascular resistance, and it will provide essential data with regard to the immunophenotype of immune injury in human lungs undergoing EVLP and selective NET removal. This will enable development of this platform for advanced reconditioning techniques underpinning multiple follow-on projects aimed at understanding the biology of lung injury. This approach will allow novel therapeutic interventions to repair organ injury to further increase the donor pool and facilitate protective immune priming of the lung for reduction in CLAD after lung transplant improving recipients’ outcome.

## 6. Technical Aspects of Selective NET Removal

Human donor lungs that have been initially assessed for the specific purpose of clinical transplantation and subsequently found to be unsuitable for direct transplantation, however suitable for conventional reconditioning using normothermic EVLP according to current widely used protocols, or even too marginal for any clinical purpose, may be suitable for this novel reconditioning approach. Prior to reconditioning, these organs will be treated in the same way as the organs used for the conventional EVLP process. Also, the surgical approach to connect the donor lungs to the EVLP circuit will remain the same as used previously. The following options to implement selective NET removal were described in animal research and can potentially be translated to human research and eventually clinical use in the future [13].

**Figure 1:**
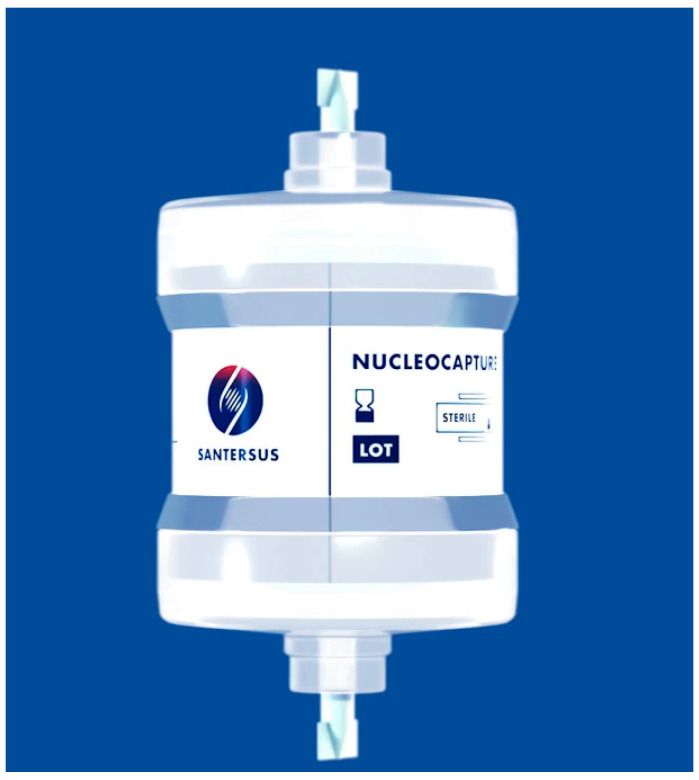
NucleoCapture Device apheresis device for selective extracorporeal removal of NETs from blood (Santersus AG, Switzerland) based on biocompatible, highly porous polymer beads conjugated with proprietary human recombinant histone H1.3 protein.

Apheresis as a treatment strategy has already been studied in several pathologies and conditions associated with NETs [17,18,25]. The aim of the future projects will be to use this established technology for the selective removal of NETs, alongside other interventions deemed to have potential to improve donor lungs during EVLP. Selective removal can be performed using a NucleoCapture apheresis device (Santersus AG, Zürich, Switzerland), which showed promising results in EVLP resuscitation in animal studies. This selective NET removal technology was initially developed as a unique strategy for the treatment of patients in a variety of therapeutic areas (Figure 1). This blood purification technology is based on biocompatible, highly porous polymer beads conjugated with proprietary human recombinant histone H1.3 protein. Histone H1.3 protein was developed by nature to be the ultimate human DNA-binding and compacting protein and is a potent sensor and effector of the ancient innate immune defense system with single-digit nanomolar DNA binding constants. It was shown that a single pass of NET-contaminated blood through the NucleoCapture device resulted in over 95% removal of NETs. In contrast to alternative pharmacological interventions targeting NETs, this technology allows for safe removal of NETs without compromising the defensive function of neutrophils.

**Figure 2:**
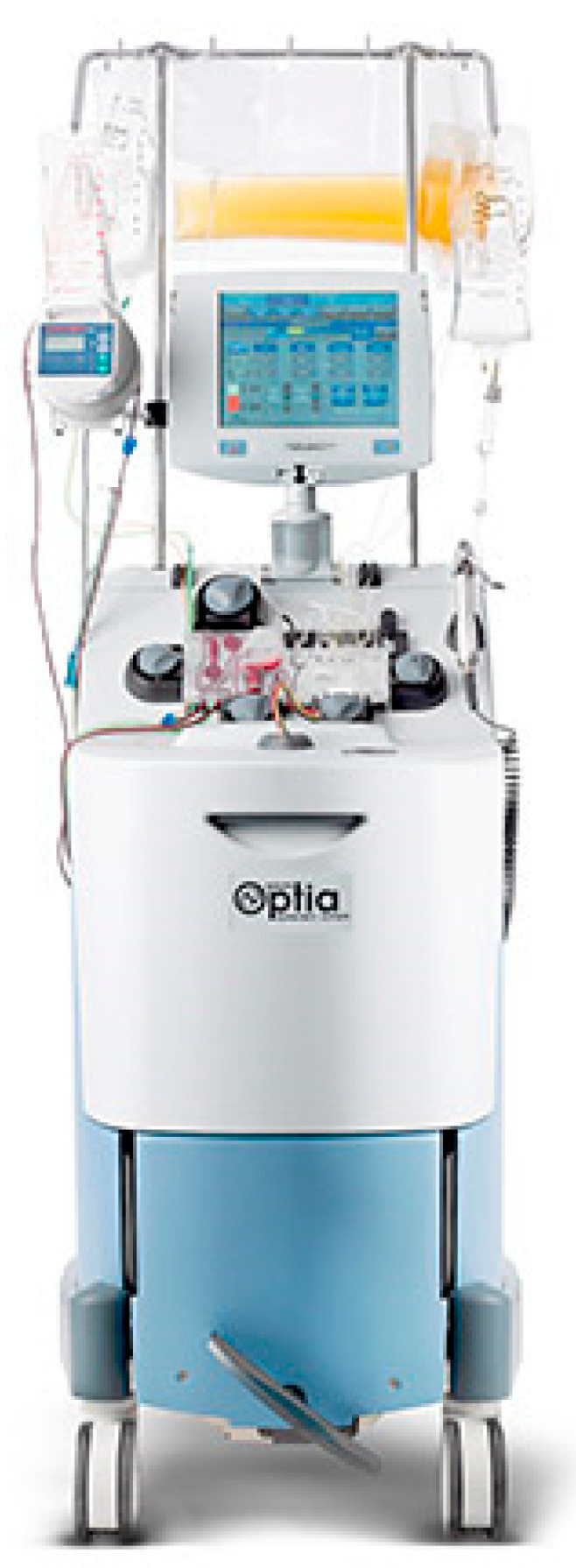
Spectra Optia Apheresis System (Terumo BCT Inc., Terumo BCT Europe NV, Zaventem, Belgium).

A NucleoCapture column can be directly connected to EVLP through a veno-venous shunt delivering perfusate from the reservoir. However, an alternative and more controlled way is to connect the NucleoCapture column to a Spectra Optia Apheresis System (perfusate flow 70 mL/min; Terumo BCT Inc., Terumo BCT Europe NV, Zaventem, Belgium) as an adjunct system in a Secondary Plasma Device (SPD) mode (Figure 2). The Spectra Optia system is an apheresis, cell processing and cell collection platform that uses continuous-flow centrifugation and optical detection technology, providing the ability to perform a wide variety of apheresis procedures and quick data processing on a single platform (Figure 2). The main benefits of this system for this research include the Automated Interface Management (AIM) System. This selective NET removal technology will allow for binding and clearance of NETs, nucleosomes, and other cell-free histones and DNA from the perfusate fluid without affecting the EVLP run.

**Figure 3:**
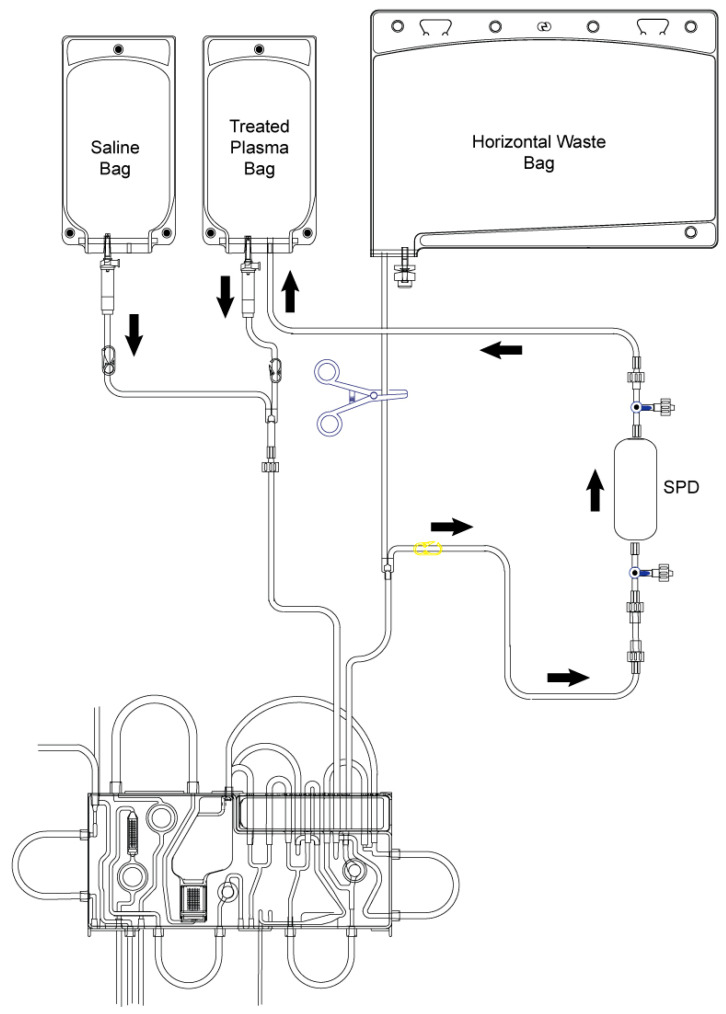
Schematic presentation of Spectra Optia Apheresis System in SPD mode (source: www.terumobct.com, accessed on 17 August 2025).

The use of the Spectra Optia Apheresis System in SPD mode, in general, allows for processing plasma through columns, filters, and secondary processing systems (Figure 3). In our case, this adjunct system can be used for processing perfusate through the NucleoCapture column. The main benefits of the Spectra Optia Apheresis System and SPD mode are adjustable flow rates to accommodate a range of devices, monitoring and display of pressure readings for the SPD with a pressure sensor, secondary processing with a mean plasma removal efficiency of 87 ± 3% and the option to pause the system for added flexibility. This dynamic technology delivers consistent, predictable results when performing plasma (perfusate) exchange with an SPD. The typical extracorporeal volume (ECV) accounts for 141 mL, whereas the maximum ECV accounts for 185 mL. Under normal operating conditions, the ECV will not exceed the typical ECV value. Under certain infrequent alarm conditions, such as during reservoir recovery after a reservoir alarm, the ECV may momentarily increase to the maximum ECV value. As SPD may be used with flow rates from 10 mL/min to 100 mL/min on the Spectra Optia system, it will be sufficient for the intended perfusate flow (70 mL/min).

### Ventilation Parameters on EVLP

Mechanical ventilation will be applied to the donor lungs in the volume-controlled ventilation setting, with peak inspiratory pressure (PIP), respiratory rate, and fraction of inspired oxygen (FiO_s_) adjusted according to clinical demands. This ventilation mode is used for conventional EVLP reconditioning, and no changes are to be made to the standard protocol.

## 7. Translation into Clinical Practice

**Figure 4:**
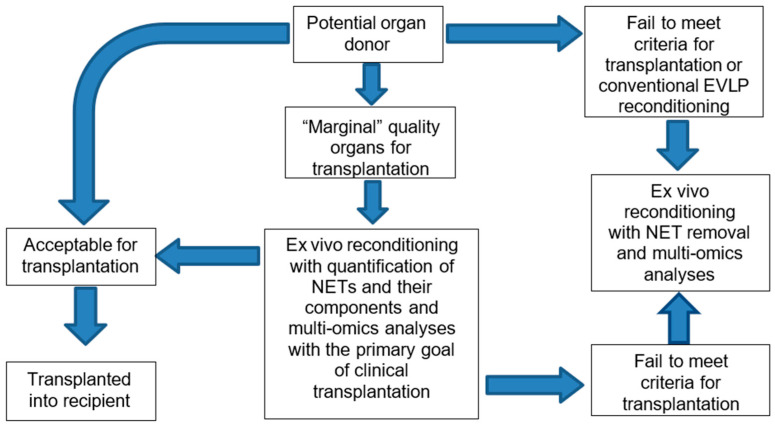
“Clinical roadmap” visualizing the translation from preclinical conceptual studies towards clinical use.

As the use of the NET removal technique in clinical practice has to be safe, efficient and justified by preclinical experimental research, several steps have to be completed regarding optimization of NET detection and removal technologies. Whereas NET quantification with subsequent multi-omics analyses using small perfusate samples may be performed within conventional EVLP using human lungs intended for subsequent clinical transplantation, active NET removal can only be applied to discarded lungs. Figure 4 summarizes a “clinical roadmap” visualizing the translational journey from the concept of NET removal, through first-in-human studies on NET detection and quantification without any therapeutic impact on donor organs as well as preclinical studies on therapeutic NET removal in human discarded organs, toward clinical implementation in the future. Only after sufficient evidence on safety and efficacy of NET quantification and therapeutic removal in a preclinical setting can such a therapeutic strategy be approved for broad use in clinical transplantation.

## 8. Safety, Regulatory and Ethical Considerations

As this technology approaches clinical reality in the near future, several safety, regulatory and ethical issues should be highlighted to allow for broad clinical use of NET removal in conjunction with conventional EVLP techniques. As all past and current research has been limited to experimental animal studies and studies using discarded human lungs, there have been no ethical issues regarding potential harm to transplant recipients. All projects involving human lungs that are intended for clinical transplantation are not subjected to therapeutic NET removal, whereas only small perfusate samples can be taken for further detection and quantification of NET in a laboratory setting. All potential adverse effects can also be ruled out due to the nature of the projects: all studies are restricted to ex vivo work on tissue that has already been removed from the body on clinical grounds; there are no changes to the routine care of either the organ donor or the organ recipient; only completely discarded organs neither suitable for direct transplantation nor suitable for potential clinical use after conventional EVLP reconditioning process are subjected to therapeutic NET removal. Moreover, it is established clinical practice in transplantation to preserve the anonymity of deceased organ donors that is also used in research. All regulatory requirements will have to be fulfilled after sufficient evidence from past and current projects on NET removal technology to facilitate clinical in-human trials in the future.

## 9. Translation to Other Organs in Transplantation

Many groups have been extensively working on similar research, assessing the performance of the NucleoCapture column in removing perfusate nucleosomes/NET, free histone and cell-free DNA (cfDNA) as well as improvements in perfusion parameters, functional state and histological features in donated livers in the setting of normothermic machine perfusion (NMP) occurring ex situ. In an animal model (12 extended criteria pig livers), they were able to show that NucleoCapture effectively removed circulating nucleosomes/NETs from the perfusate during NMP, improving graft function and mitigating ex situ reperfusion injury (ERI) [21]. In another research project involving 10 extended criteria and discarded human livers, it was demonstrated that high circulating NET suggested the need for mitigating potential complications associated with transplantation through application of novel extracorporeal columns, such as NucleoCapture [26]. The efficiency of the NucleoCapture column in reducing extracellular histones was also shown in another animal research model involving 10 porcine livers during normothermic machine perfusion using both leucodepleted and whole blood [27,28,29,30]. As normothermic perfusion machines in organ transplantation have similar perfusion characteristics, translation of the approach to other organs will play a major role in improving the utilization rate [31]. While there have been no in-human trials on NET removal in transplantation research on other organs, there is increasing evidence on the high potential of this technology also in liver and kidney transplantation [32,33,34]. Moore et al. showed that reducing cell-free DNA and NET may improve outcomes of orthotopic liver transplantation based on their involvement in the coagulation process and reduced thrombotic complications after transplant [35]. Apart from the negative impact of NET on the coagulation cascade, NETs are highly involved in the acute rejection process after liver transplantation. In this respect, not physical removal but inhibiting NET formation using diphenyleneiodonium that suppresses activation of the NADPH/ROS/PAD4 signaling pathway was shown to be an alternative way in liver transplantation [36]. Similarly, Liu et al. showed that tetramethylpyrazine may also inhibit NET formation and alleviate hepatic ischemia–reperfusion injury in rat liver transplantation [37]. Such approaches may successfully be translated to other organs including lungs as mechanisms of rejection, which seem to have significant correlation of mechanisms and pathways. In corroboration of this evidence, it was not surprising that recent evidence supported amelioration of ischemia–reperfusion injury after orthotopic heart transplantation using suppressing mechanisms on NET formation. Tian et al. described inhibition of macrophage ARID3A as a tool to alleviate myocardial ischemia–reperfusion injury after heart transplantation by reducing THBS1/CD47 signaling-mediated NET formation [38]. Similarly, there is intensive research on identification of potential targets regulating NET and their detrimental effect in acute rejection of kidney transplantation based on transcriptomics and animal experiments [39].

## 10. Conclusions

This article describes the current state of research on the role of NETs in inflammatory conditions and in allograft injury during donation. Previous evidence showed that allograft function can be significantly improved with selective NET removal along with conventional EVLP resuscitation. However, translation of this evidence into human research is necessary to prove efficiency of this technique for potential clinical implementation. We summarized long-term experimental research perspectives and potential clinical use of this strategy in the future. Several multiple omics analyses such as genomics, transcriptomics, immunomics and proteomics will be the main focus of allograft assessment in the future.

## Figures and Tables

**Table 1:** Overview of experimental studies on EVLP.

Platform	Outcome
Extended preservation	Prolonged preservation feasible using optimization of perfusion and ventilation strategies and prone positioning
Marginal donors	Use of A2A receptor antagonists, hydrogen sulfide, methylprednisolone and perfluorocarbon-based oxygen carriers (PFCOCs) improves outcome
Specific lung injury	Use of antibiotics (sepsis), surfactant (aspiration), ultraviolet C and photodynamic therapy (hepatitis C) could restore lung function
Dual EVLP	Use of antibiotics (sepsis), surfactant (aspiration), ultraviolet C and photodynamic therapy (hepatitis C) could restore lung function
EVLP biomarkers	Cytokines, cell death, and endothelial-related molecules may predict survival and be used to monitor various therapeutics for donor lung repair
Therapy delivery	IL-10 overexpression delivered via an adenovirus vector and silencing of Fas using small interfering RNA to reduce IRI and inflammation, respectively

IRI, ischemia–reperfusion injury.
